# Association between an oncology psychology course and caring ability improvements in postgraduate medical students

**DOI:** 10.3389/fpsyg.2025.1714423

**Published:** 2025-11-27

**Authors:** Xin Hua, Lin Xiao, Lu Wu, Jie-Wen Chen, Sha-Sha Du, De-Huan Xie, Heng-Wen Sun

**Affiliations:** 1 Department of Radiation Oncology, Guangdong Provincial People's Hospital (Guangdong Academy of Medical Sciences), Southern Medical University, Guangzhou, China; 2 School of Education, Jiangxi Normal University, Nanchang, China

**Keywords:** oncology, psychology, students, empathy, medical education

## Abstract

**Background:**

Cancer patients experience significant psychological distress that requires healthcare providers with well-developed caring abilities. Medical education traditionally underemphasizes humanistic competencies, particularly in oncology contexts. This study aimed to evaluate the impact of a specialized oncology psychology course on the caring ability of postgraduate medical students specializing in clinical medicine.

**Methods:**

A quasi-experimental pre-post design was used with a sample of 32 postgraduate medical students who completed both pre- and post-course assessments. The study was conducted at South China University of Technology in Guangzhou, from September 2024 to January 2025. The oncology psychology course incorporating theoretical foundations, psychological assessment, intervention strategies, and clinical applications through multimodal pedagogy including lectures, case discussions, standardized patient simulations, and clinical observations. The Caring Ability Inventory (CAI) was used to measure caring ability across three dimensions (Knowing, Courage, and Patience) before and after the course. Paired *t-*tests and independent samples *t-*tests were employed to analyze data using SPSS version 23.0.

**Results:**

A total of 32 students completed both assessments. The total CAI score increased significantly from pre-course to post-course (188.53 ± 20.74 vs. 197.53 ± 22.75, *P* < 0.001). Significant improvements were observed in all three dimensions: Knowing (71.72 ± 8.95 vs. 75.97 ± 10.15, *P* < 0.001), Courage (57.69 ± 10.50 vs. 60.31 ± 10.71, *P* < 0.001), and Patience (59.13 ± 5.34 vs. 61.30 ± 5.47, *P* < 0.001). Female students demonstrated consistently higher Patience dimension scores than males both before and after the course (*P* < 0.05). Student leaders showed superior total CAI and Courage dimension scores compared to non-leaders at both time points (*P* < 0.05).

**Conclusions:**

The oncology psychology course was associated with significant improvements in caring ability among postgraduate medical students across all dimensions. The findings support the integration of specialized psycho-oncology curricula in medical education and highlight the importance of considering demographic factors in educational design. This evidence-based approach to developing caring competencies addresses critical gaps in preparing future oncologists for patient-centered care.

## Introduction

Cancer represents one of the leading causes of morbidity and mortality worldwide, with an estimated 19.3 million new cases diagnosed annually (Sung et al., 2021). Beyond the physical burden of disease, cancer patients frequently experience significant psychological distress, including anxiety, depression, and fear of death, which adversely affect treatment adherence, quality of life, and clinical outcomes (Caruso et al., 2017; Park et al., 2018). The prevalence of clinically significant anxiety and depression among cancer patients ranges from 20% to 40%, substantially higher than in the general population (Mehnert et al., 2014).

Given the complex psychological needs of cancer patients, healthcare professionals require not only clinical expertise but also well-developed caring abilities, encompassing empathetic communication, psychological assessment skills, and the capacity to provide emotional support. Caring ability is a core competency for healthcare professionals, defined as the capacity to recognize, respond to, and alleviate patients' physical, emotional, and psychological needs—particularly critical in oncology, where patients face unique distress from diagnosis, treatment, and prognosis uncertainty (Woodward, 1998). Watson's Theory of Human Caring (Watson, 2008) frames caring as a “transpersonal” process that transcends technical medical skills to prioritize human connection, empathy, and holistic patient well-being. The theory identifies 10 carative factors, including “developing a helping-trusting relationship,” “promoting expression of positive and negative feelings,” and “using creative problem-solving for caring processes”—all directly aligned with the CAI's three dimensions (Nkongho, 2003). In oncology settings, Watson's theory emphasizes that caring is not just a “skill” but a moral obligation to address the “whole person”—a principle underscored by rising evidence that caring-oriented communication reduces cancer patients' psychological distress and improves treatment adherence (Labrague et al., 2020). This theoretical link justifies our focus on enhancing caring ability via an oncology-specific course: without intentional training, postgraduate medical students may prioritize technical skills (e.g., tumor staging) over the transpersonal caring processes Watson highlights.

Medical education traditionally emphasizes biomedical knowledge and technical skills while underemphasizing humanistic competencies and psychological care skills (Levinson et al., 1997). This educational gap is particularly problematic in oncology, where the psychological complexity of patient care demands enhanced caring abilities from healthcare providers. The Association of American Medical Colleges has recognized this need by identifying “interpersonal and communication skills” and “professionalism” as core competencies for medical graduates.

International medical curricula have increasingly incorporated psychosocial oncology and patient-centered care training. Studies have demonstrated that structured educational interventions can significantly enhance healthcare students' caring abilities and empathetic responses. Brunero et al. reported that undergraduate nursing students showed improved caring behaviors following a mental health clinical placement program (Brunero et al., 2010). Similarly, Williams et al. found that medical students' empathy scores increased significantly after completing a patient-centered communication course (Williams et al., 2014).

In China, recent medical education reforms have emphasized the integration of humanistic education into clinical training programs. The National Health Commission has mandated that medical schools strengthen medical humanities education to cultivate students' professional ethics and caring abilities. However, empirical research on the effectiveness of specialized psychological oncology courses remains limited. Most existing studies have focused on general medical psychology courses or undergraduate populations, with insufficient attention to postgraduate medical students who directly engage in clinical oncology practice (Kosik et al., 2018; Zhang et al., 2023).

Oncology psychology courses, which integrate knowledge of cancer-related psychological phenomena with practical intervention strategies, represent a targeted approach to enhancing caring abilities in future oncologists. These specialized curricula address the unique psychological challenges faced by cancer patients, including adjustment disorders, anticipatory anxiety, and existential concerns (Dekker et al., 2020). Caring ability—measured via the CAI's Knowing (recognize needs), Courage (act on intentions), and Patience (tolerate distress) dimensions—aligns with but is distinct from core psychological constructs critical to oncology care. Empathy (share/understand emotions) serves as its precursor: it enables the CAI's “Knowing” dimension (e.g., sensing a patient's unspoken fear) but does not equate to caring ability's active, behavioral focus (e.g., addressing that fear via the “Courage” dimension). Compassion (desire to relieve suffering) provides its motivational core: it drives the “Patience” dimension (remaining present during patient grief) by pairing concern with commitment to act. Emotional intelligence (perceive/regulate emotions) acts as its foundational skill set: it supports “Knowing” (interpreting emotional cues), “Patience” (managing provider distress), and “Courage” (using empathy to guide support)—critical for avoiding compassion fatigue in oncology. This distinction justifies our course's focus: postgraduate students often have empathy/compassion but need training to translate these into sustained caring ability, as our CAI-based evaluation aims to capture.

The present study addresses two significant gaps in the current literature: first, the limited research on caring ability development among postgraduate medical students specializing in clinical medicine; and second, the lack of evaluation of specialized oncology psychology curricula on caring ability outcomes. Thus, this study's aim—to evaluate the impact of an Oncology Psychology course on self-perceived caring ability among postgraduate medical students in southern China—addresses unmet needs in both global and Chinese literature, with findings that are uniquely applicable to clinical oncology training for Chinese postgraduate medical students.

## Methods

### Study design and setting

This study employed a quasi-experimental pre-post design to evaluate the effectiveness of the Oncology Psychology course on caring ability among postgraduate medical students. The study was conducted at South China University of Technology in southern China between September 2024 and January 2025. All participants provided written informed consent prior to enrollment.

### Participants

Participants were recruited from postgraduate medical students specializing in clinical medicine who enrolled in the Oncology Psychology course during the 2024–2025 academic year. Prior to recruitment, all eligible students received a written “Study Information Sheet” that clearly stated the study's purpose, required time commitment (20–25 min for pre/post surveys), and right to withdraw. Only students who provided written informed consent were included in the study. Two students declined participation (1 due to time constraints, 1 for unspecified reasons) and were excluded from all analyses. A total of 34 students were initially recruited, with 32 completing both pre- and post-course assessments, resulting in a response rate of 94.1%. Inclusion criteria: (1) enrolled as full-time postgraduate medical students specializing in clinical medicine; (2) completed all course modules and assessments; (3) had no prior formal training in psycho-oncology or oncology psychology. Exclusion criteria: (1) course attendance rate below 80%; (2) incomplete questionnaire responses (>10% missing items); (3) concurrent enrollment in other psychology or communication skills courses; (4) history of psychiatric disorders requiring treatment.

### Oncology psychology course

**Figure 1:**
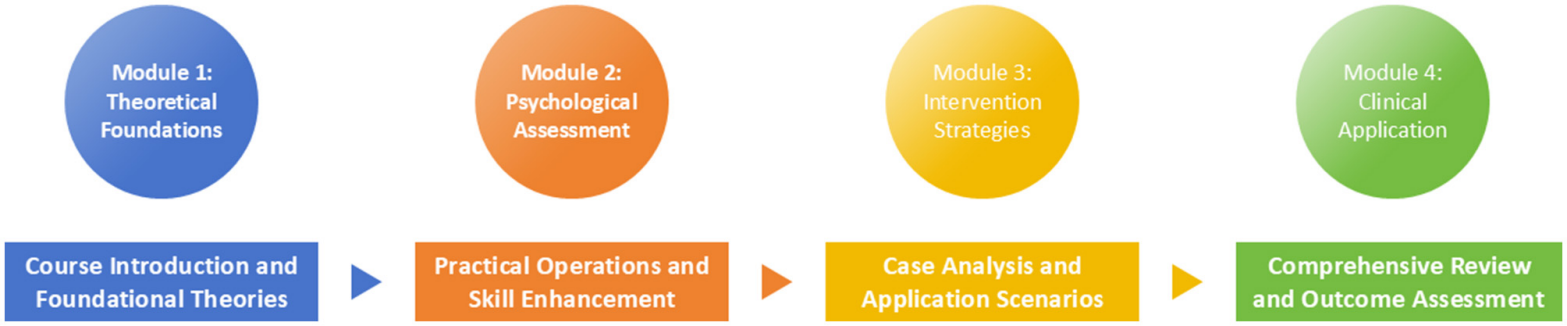
Diagram of the oncology psychology course structure.

The course content was systematically developed based on evidence-based psycho-oncology literatures and validated educational frameworks to ensure scientific rigor and clinical relevance. Prior to course delivery, the 4-member instructor team completed a 4-h standardized training program to ensure uniform delivery of course content and alignment with psycho-oncology educational best practices. The course comprised 24 contact hours (distributed evenly across the 6-week period, 4 h/week) delivered through multiple pedagogical approaches. As summarized in Figure 1, the course followed a “knowledge → skill → application” logic, was structured into four sequential modules:

Module 1: Theoretical Foundations (6 h, delivered in 2 weekly sessions, 3 h/session over weeks 1–2) covered the bidirectional relationship between psychological factors and cancer outcomes, prevalence of psychological distress in cancer populations, and theoretical models of adjustment to cancer diagnosis.

Module 2: Psychological Assessment (6 h, delivered in 2 weekly sessions, 3 h/session over weeks 2–3) included training in validated screening instruments such as the Hospital Anxiety and Depression Scale (HADS), Distress Thermometer, and structured clinical interview techniques for identifying psychological distress in cancer patients.

Module 3: Intervention Strategies (8 h, delivered in 4 biweekly sessions, 2 h/session over weeks 3–5, 2-week duration, with 3-day intervals between sessions to allow students to practice skill drills between classes) focused on evidence-based psychological interventions including cognitive-behavioral therapy techniques, mindfulness-based interventions, and supportive-expressive therapy approaches tailored for different phases of the cancer trajectory.

Module 4: Clinical Application (4 h, delivered in 1 intensive week, including 2 h of standardized patient simulations and 2 h of clinical observations-with a 2-day interval between simulation and observation to debrief and refine skills) involved supervised clinical observations in the oncology department, standardized patient encounters, and case-based learning sessions using actual patient scenarios (with appropriate ethical clearance).

Teaching methods were designed to promote active learning and skill acquisition. Didactic lectures (40% of contact time) were delivered by certified oncologists and clinical psychologists using multimedia presentations and video vignettes. Small group case discussions (30% of contact time) involved analysis of de-identified patient cases in groups of 6–8 students, facilitated by faculty members. Standardized patient simulations (20% of contact time) provided experiential learning opportunities with trained actors portraying cancer patients experiencing various psychological challenges. Clinical observations (10% of contact time) were conducted in the hospital oncology unit under direct supervision of attending physicians. Course assessment was designed to evaluate knowledge acquisition: Case Analysis Assignments (50%): after Module 3 (Intervention Strategies), students submitted an analysis of a de-identified cancer patient case (e.g., a 45-year-old breast cancer patient with treatment-related anxiety). Assignments required identifying psychological needs, selecting evidence-based interventions, and justifying choices with course content (e.g., cognitive-behavioral therapy techniques). Simulation Performance (50%): during Module 4 (Clinical Application), students participated in 2 standardized patient (SP) simulations (30 min each). Trained SPs portrayed patients with cancer-related distress (e.g., fear of recurrence, family conflict), and students were evaluated on their ability to: (a) recognize psychological needs, (b) use empathetic communication, and (c) propose initial intervention plans. Evaluations were conducted via video recording, with feedback provided within 48 hours. The 4-member instructor team (1 clinical psychologist, 3 certified oncologist) collaborated closely to ensure integration of psychological and clinical oncology perspectives. Prior to the course, the psychologist and oncologist jointly developed Module 3 (Intervention Strategies) to ensure interventions were: (a) evidence-based in psychology (e.g., mindfulness for distress) and (b) clinically feasible in oncology settings (e.g., adapting CBT to 15-min clinic visits). For example, they co-created a “quick-reference guide” for students, linking common patient concerns (e.g., chemo-related anxiety) to 5-min supportive interventions. 40% of contact hours (e.g., Module 2′s assessment training, Module 4′s simulation debriefs) were co-facilitated by the psychologist and oncologist. During simulation debriefs, the oncologist provided clinical context (e.g., “This patient's fear of recurrence aligns with 30% of Stage II breast cancer patients we see”), while the psychologist guided discussion on emotional validation techniques (e.g., “How might you acknowledge their fear without minimizing it?”).

### Outcome measures

Caring ability was measured using the Caring Ability Inventory (CAI), originally developed by Nkongho (2003) and validated in Chinese version (Hu et al., 2022; Xu, 2008), with strong internal consistency reliability of total scale: Cronbach's α = 0.84; Knowing dimension: Cronbach's α = 0.81; Courage dimension: Cronbach's α = 0.70; Patience dimension: Cronbach's α = 0.74. The CAI comprises 37 items distributed across three dimensions: Knowing (14 items, score range 14–98) assesses cognitive awareness of others' needs; Courage (13 items, score range 13–91) measures willingness to act despite risks; and Patience (10 items, score range 10–70) evaluates tolerance and perseverance in caring relationships. Items are rated on a 7-point Likert scale (1 = strongly disagree to 7 = strongly agree), with 13 reverse-scored items. Total scores range from 37 to 259, with higher scores indicating greater caring ability. For the current study sample, we calculated the internal consistency reliability of the CAI using Cronbach's α coefficient to verify its psychometric suitability, Total CAI scale: Cronbach's α = 0.87; Knowing dimension: Cronbach's α = 0.83; Courage dimension: Cronbach's α = 0.81; Patience dimension: Cronbach's α = 0.79. These values indicate good to excellent internal consistency for the CAI in our sample.

### Data collection procedures

Data collection followed standardized protocols to ensure consistency and minimize self-report biases and protect participant privacy, with targeted measures as follows: (1). Anonymity Guarantees, to eliminate concerns about judgment or retaliation that could skew self-reports, full anonymity was enforced throughout data collection. Participants did not provide personal identifiers (e.g., full name, student ID) on CAI questionnaires. Instead, each participant created a unique, non-identifiable code to link their pre- and post-course responses. This code was not stored with any personal information and was permanently deleted after data matching. All questionnaire data (digital files via Wenjuanxing platform) were stored in a password-protected database, with access limited to core research team members (independent of course instruction). Course instructors and university administrators had no access to individual responses—only group-level summary data were shared for course evaluation. (2). Response Bias Mitigation, several strategies were implemented to reduce common self-report biases. Research assistants who administered the CAI were unaware of the study's primary hypothesis. They provided neutral instructions: “Please answer based on your actual experiences, not what you think is ‘expected'—there are no right or wrong answers.” This minimized demand characteristics (participants altering responses to align with perceived study goals). Questionnaires with >10% missing items or identical responses to all items were excluded. This removed data from participants who completed the survey carelessly, reducing noise from low-quality self-reports. Baseline assessment (T1) occurred during the first week of the course before any content delivery. Follow-up assessment (T2) was conducted in the final week after course completion. Online questionnaires were administered through a secure web-based platform (Wenjuanxing: www.wjx.cn) with unique participant identifiers to enable matched pair analysis while maintaining anonymity. Research assistants, blinded to study hypotheses, supervised data collection and provided technical support as needed.

### Statistical analysis

Descriptive statistics included frequencies and percentages for categorical variables and means with standard deviations for continuous variables. Prior to conducting parametric tests, the normality of continuous data (CAI total and dimension scores at pre- and post-course) was formally tested using Shapiro-Wilk Tests and Q-Q plots (quantile-quantile plots). Paired-samples *t-*tests were used to compare pre- and post-course CAI total and dimension scores. Independent Samples *t-*tests were used to analyze differences in CAI scores across demographic subgroups. Cohen's d was computed, and interpretations follow conventional thresholds: |d| < 0.2 (trivial), 0.2 ≤ |d| < 0.5 (small), 0.5 ≤ |d| < 0.8 (medium), |d|≥0.8 (large). For the 3 pre-hypothesized subgroup-outcome pairs (gender-Patience, grade-Courage, student leadership-Total/Courage), we applied the Bonferroni correction to adjust the significance threshold. Statistical significance was set at α = 0.05, with 95% confidence intervals reported for all estimates. Statistical analyses were performed using SPSS version 23.0 (IBM Corp., Armonk, NY).

## Results

### Participant characteristics

A total of 32 postgraduate medical students were included in the final analysis. The demographic characteristics of the participants are presented in Table 1. The majority were aged 24–25 years (78.1%), with an equal gender distribution (50% male, 50% female). Most were in their third year of postgraduate training (71.9%), did not hold student leadership positions (75.0%), and were from urban areas (59.4%). Slightly more than half of the participants (53.1%) reported having siblings.

**Table 1:** Demographic characteristics of students.

**Characteristic**	***N* (*n* %)**
**Age**	
23 years	1 (3.1%)
24 years	11 (34.4%)
25 years	14 (43.8%)
26 years	4 (12.5%)
27 years	2 (6.3%)
**Gender**	
Male	16 (50.0%)
Female	16 (50.0%)
**Grade**	
Grade 2	9 (28.1%)
Grade 3	23 (71.9%)
**Student leader**	
Yes	8 (25.0%)
No	24 (75.0%)
**Residence**	
Urban	19 (59.4%)
Rural	13 (40.6%)
**Has sibling or not**	
Yes	17 (53.1%)
No	15 (46.9%)

### Effects of the oncology psychology course on caring ability

The total CAI score and scores in all three dimensions (Knowing, Courage, and Patience) increased significantly from pre- to post-course assessments (all *P* < 0.001, Table 2). The total CAI score increased by an average of 9.00 points after the course, with a medium effect size (Cohen's d = 0.78), indicating a practically meaningful enhancement in overall caring ability. Among dimensions: the Knowing dimension showed a medium effect size (Cohen's d = 0.65), reflecting substantial gains in students' ability to recognize cancer patients' psychological needs; the Courage dimension had a small-to-medium effect size (Cohen's d = 0.49), consistent with modest but meaningful improvements in students' confidence to provide psychological support; the Patience dimension demonstrated a medium effect size (Cohen's d = 0.62), highlighting enhanced tolerance for emotional expressions during patient interactions. The largest relative increase was observed in the Courage dimension (4.54%), followed by the Knowing (5.93%) and Patience (3.67%) dimensions.

**Table 2:** Comparison of CAI scores before and after course (Mean ± SD).

**Characteristic**	**Before**	**After**	***t***	***P***	**Cohen's d**
Total score	188.53 ± 20.74	197.53 ± 22.75	8.47	< 0.001	0.78
Knowing	71.72 ± 8.95	75.97 ± 10.15	7.30	< 0.001	0.65
Courage	57.69 ± 10.50	60.31 ± 10.71	4.28	< 0.001	0.49
Patience	59.13 ± 5.34	61.3 ± 5.47	7.00	< 0.001	0.62

### Subgroup analyses of CAI scores

No significant differences were found in total CAI scores or dimension scores between the younger (< 25 years) and older (≥25 years) age groups, either before or after the course (all *P* > 0.05, Tables 3, 4). Female students scored significantly higher than male students in the Patience dimension, both before (*P* = 0.018) and after (*P* = 0.009) the course. No significant gender differences were observed in total CAI scores or the Knowing and Courage dimensions (all *P* > 0.05, Tables 3, 4). Grade 2 students had a significantly higher score in the Courage dimension compared to Grade 3 students after the course (*P* = 0.049). No other significant differences were found in total CAI scores or the other dimension scores between grade levels (all *P* > 0.05, Tables 3, 4). Student leaders had significantly higher total CAI scores than non-leaders, both before (*P* = 0.027) and after (*P* = 0.015) the course. They also scored higher in the Courage dimension, both before (*P* = 0.011) and after (*P* = 0.007) the course, as shown in Tables 3, 4. No significant differences were found in total CAI scores or dimension scores between students from urban vs. rural areas, or between those with and without siblings (all *P* > 0.05, Tables 3, 4).

**Table 3:** Comparison of CAI scores in different subgroups before course (Mean ± SD).

**Variables**	**Numbers**	**Total score**	**Knowing**	**Courage**	**Patience**
**Age**					
< 25 years	12	191.58 ± 21.73	73.00 ± 10.85	60.58 ± 10.26	58.00 ± 5.08
≥ 25 years	20	186.70 ± 20.50	70.95 ± 7.80	55.95 ± 10.52	59.80 ± 5.51
Δ		−4.88 ± 21.10	−2.05 ± 9.35	−4.63 ± 10.39	1.80 ± 5.29
*t*		0.64	0.57	1.22	−0.92
*P*		0.528	0.575	0.233	0.365
**Gender**					
Male	16	185.31 ± 19.85	70.81 ± 9.30	57.56 ± 10.07	56.94 ± 5.00
Female	16	191.75 ± 21.78	72.63 ± 8.79	57.81 ± 11.26	61.31 ± 4.88
Δ		−6.44 ± 20.76	−1.82 ± 9.04	−0.25 ± 10.65	−4.37 ± 4.94
*t*		−0.87	−0.57	−0.07	−2.50
*P*		0.389	0.575	0.948	0.018
**Grade**					
Grade 2	19	191.53 ± 22.89	72.68 ± 10.27	60.16 ± 10.79	58.68 ± 5.75
Grade 3	13	184.15 ± 17.10	70.31 ± 6.70	54.08 ± 9.30	59.77 ± 4.85
Δ		7.38 ± 19.99	2.37 ± 8.58	6.08 ± 10.04	−1.09 ± 5.30
*t*		0.99	0.79	1.65	−0.56
*P*		0.332	0.435	0.109	0.581
**Student leader**					
Yes	8	202.38 ± 20.28	75.63 ± 7.41	65.63 ± 9.16	61.13 ± 6.51
No	24	183.92 ± 19.15	70.42 ± 9.17	55.04 ± 9.69	58.46 ± 4.87
Δ		18.46 ± 19.71	5.21 ± 8.30	10.59 ± 9.42	2.67 ± 5.70
*t*		2.33	1.45	2.71	1.23
*P*		0.027	0.157	0.011	0.227
**Residence**					
Urban	19	186.68 ± 23.36	70.32 ± 9.53	57.16 ± 11.97	59.21 ± 5.94
Rural	13	191.23 ± 16.77	73.77 ± 7.93	58.46 ± 8.31	59.00 ± 4.56
Δ		−4.55 ± 20.06	−3.45 ± 8.73	−0.70 ± 10.14	0.21 ± 5.25
*t*		−0.60	−1.08	−0.34	0.11
*P*		0.552	0.291	0.736	0.915
**Has sibling or not**					
Yes	15	188.13 ± 23.73	72.13 ± 10.34	57.07 ± 12.79	58.93 ± 6.25
No	17	188.88 ± 18.49	71.35 ± 7.83	58.24 ± 8.37	59.29 ± 4.59
Δ		−0.75 ± 21.11	0.78 ± 9.09	−1.17 ± 10.58	−0.36 ± 5.42
*t*		−0.10	0.24	−0.31	−0.19
*P*		0.921	0.810	0.759	0.852

**Table 4:** Comparison of CAI scores in different subgroups after course (Mean ± SD).

**Variables**	**Numbers**	**Total score**	**Knowing**	**Courage**	**Patience**
**Age**					
< 25 years	12	201.58 ± 25.89	77.33 ± 12.94	63.50 ± 10.15	60.75 ± 5.79
≥25 years	20	195.10 ± 20.97	75.15 ± 8.31	58.40 ± 10.83	61.55 ± 5.40
Δ		6.48 ± 23.40	2.18 ± 10.63	5.10 ± 10.49	−0.80 ± 5.59
*t*		0.78	0.52	1.32	−0.40
*P*		0.444	0.608	0.197	0.696
**Gender**					
Male	16	193.88 ± 19.79	75.06 ± 10.11	60.00 ± 8.69	58.81 ± 5.13
Female	16	201.19 ± 25.48	76.88 ± 10.43	60.63 ± 12.70	63.69 ± 4.78
Δ		−7.31 ± 22.63	−1.82 ± 10.27	−0.63 ± 10.74	−4.88 ± 4.95
*t*		−0.91	−0.50	−0.16	−2.78
*P*		0.372	0.621	0.872	0.009
**Grade**					
Grade 2	19	201.37 ± 25.73	76.95 ± 12.09	63.37 ± 10.64	61.05 ± 5.71
Grade 3	13	191.92 ± 16.90	74.54 ± 6.54	55.85 ± 9.50	61.54 ± 5.32
Δ		9.45 ± 21.31	2.41 ± 9.29	7.52 ± 10.07	−0.49 ± 5.52
*t*		1.25	0.73	2.05	−0.25
*P*		0.220	0.473	0.049	0.807
**Student leader**					
Yes	8	214.00 ± 25.29	81.38 ± 9.47	68.88 ± 11.10	63.75 ± 6.94
No	24	192.04 ± 19.41	74.17 ± 9.89	57.46 ± 9.12	60.42 ± 4.77
Δ		21.96 ± 22.30	7.21 ± 9.68	11.42 ± 10.11	3.33 ± 5.87
*t*		2.57	1.84	2.91	1.52
*P*		0.015	0.089	0.007	0.138
**Residence**					
Urban	19	196.00 ± 26.60	74.74 ± 11.39	60.05 ± 12.55	61.21 ± 6.12
Rural	13	199.77 ± 16.30	77.77 ± 8.08	60.69 ± 7.73	61.31 ± 4.61
Δ		−3.77 ± 21.45	−3.03 ± 9.74	−0.64 ± 10.14	−0.10 ± 5.37
*t*		−0.45	−0.83	−0.163	−0.049
*P*		0.653	0.415	0.871	0.962
**Has sibling or not**					
Yes	15	197.27 ± 28.02	76.07 ± 12.22	60.27 ± 13.68	60.93 ± 6.60
No	17	197.76 ± 17.76	75.88 ± 8.30	60.35 ± 7.66	61.53 ± 4.45
Δ		−0.49 ± 22.89	0.19 ± 10.26	−0.08 ± 10.67	−0.60 ± 5.53
*t*		−0.061	0.050	−0.022	−0.30
*P*		0.952	0.960	0.982	0.764

## Discussion

This study demonstrated that a structured oncology psychology course was associated with improvements in caring ability among postgraduate medical students, as reflected in higher post-course CAI scores. The observed improvements align with contemporary educational frameworks emphasizing the integration of psychosocial competencies in medical training (Levinson et al., 1997). The magnitude of improvement (mean increase of 9.00 points in total CAI score) represents a clinically meaningful enhancement in caring capacity, with effect sizes consistent with previous educational interventions in healthcare professional development (Mercer and Reynolds, 2002).

The findings of this study consistent with international psycho-oncology training research. Our moderate effect sizes align with German communication skills training outcomes reported by Stiefel et al., who found similar improvements in healthcare provider self-efficacy and confidence in addressing psychological distress (Stiefel et al., 2013). The enhancement in the Knowing dimension corresponds with European Organisation for Research and Treatment of Cancer (EORTC) findings, where Razavi et al. demonstrated that structured psycho-oncology education significantly improved healthcare providers‘ knowledge of psychological assessment techniques (Razavi et al., 2002). Improvements in the Courage dimension mirror UK National Health Service training results, showing that oncology-specific communication training reduced provider anxiety about discussing psychological issues and increased willingness to engage in difficult conversations (Fallowfield et al., 2002). The Patience dimension enhancement shows interesting parallels with Asian healthcare education research, found that empathy-focused training in Japanese oncology settings improved healthcare providers' tolerance for emotional expressions (Kataoka et al., 2009). The consistently higher Patience scores among female students align with meta-analysis across 13 countries, which documented consistent gender differences in empathic responding regardless of cultural context (Thompson and Voyer, 2014).

The significant improvements observed across all CAI dimensions support the efficacy of experiential learning approaches in developing caring competencies. The enhancement in the Knowing dimension (mean increase of 4.25 points) likely reflects the course's emphasis on psycho-oncology knowledge acquisition, consistent with research demonstrating that targeted education about cancer patients' psychological needs improves healthcare providers' ability to recognize and respond to distress (Fallowfield and Jenkins, 2004; Baile et al., 2000). The improvement in the Courage dimension (mean increase of 2.62 points) aligns with findings from simulation-based education research, which demonstrates that standardized patient encounters reduce anxiety and increase confidence in addressing psychosocial issues (Cleland et al., 2009). This finding is particularly relevant given documented barriers to discussing psychological concerns in oncology settings, including provider discomfort and perceived lack of competence (Heaven et al., 2006). The enhancement in the Patience dimension (mean increase of 2.17 points) corresponds with evidence supporting communication skills training in developing empathetic responses and tolerance for emotional expressions (Duberstein et al., 2007; Stewart et al., 2000). This improvement is noteworthy given the emotional demands of oncology practice and the importance of patient-centered communication in cancer care (Epstein et al., 2007).

The consistently higher Patience dimension scores among female students align with extensive literature documenting gender differences in empathic responding and caregiving behaviors (Baron-Cohen and Wheelwright, 2004). Meta-analytic evidence suggests these differences reflect both sociocultural factors and potential neurobiological variations in empathy processing (Christov-Moore et al., 2014). However, the absence of gender differences in overall caring ability suggests that structured training can effectively develop core competencies regardless of baseline differences. The superior post-course Courage dimension scores among Grade 2 students may reflect developmental readiness and reduced competing academic demands. Research on professional identity formation suggests that earlier-stage learners may be more receptive to psychosocial skill development before clinical responsibilities become overwhelming (Cruess et al., 2014; Jarvis-Selinger et al., 2012). This finding supports optimal timing for humanities education in medical curricula. Student leaders demonstrated consistently higher total CAI scores and Courage dimension scores both pre- and post-intervention. This pattern suggests that leadership experiences may cultivate interpersonal competencies transferable to caring relationships (Avolio et al., 2009). The sustained differences post-intervention indicate that while the course benefits all students, those with prior leadership experience may have enhanced capacity for skill acquisition and application (Day et al., 2014).

These findings support several key principles for designing caring ability curricula in medical education. First, the effectiveness of multimodal pedagogy combining didactic instruction, case-based learning, simulation, and clinical observation aligns with best practices in healthcare professional education (Frenk et al., 2010). The integration of theoretical knowledge with experiential learning appears essential for developing both cognitive understanding and behavioral competencies. Second, the differential responses across demographic subgroups suggest the value of personalized educational approaches. While the course benefited all participants, targeted interventions addressing specific needs (e.g., confidence-building for students without leadership experience, patience-focused training for male students) may optimize outcomes (Eva, 2005). Third, the timing of humanities education appears critical. The superior response among Grade 2 students suggests that earlier integration of psychosocial training may be more effective than later-stage interventions when clinical demands intensify (Billings-Gagliardi et al., 2004).

The magnitude of improvement observed in this study is consistent with previous evaluations of communication skills training and empathy development programs (Brunero et al., 2010; Stepien and Baernstein, 2006). However, the specific focus on oncology psychology represents a novel contribution to the literature, addressing the recognized need for specialized training in cancer care communication (Nekhlyudov et al., 2024). The gender differences observed align with findings from nursing education research demonstrating persistent gender disparities in caring behaviors despite educational interventions (Dulay et al., 2018). However, the absence of overall gender differences in caring ability improvement suggests that well-designed curricula can promote equitable skill development.

Several research priorities emerge from these findings. Randomized controlled trials comparing the oncology psychology course to standard curricula or alternative interventions would strengthen causal inference. Multi-institutional studies with larger, more diverse samples would enhance generalizability. Longitudinal follow-up studies tracking participants into clinical practice would assess skill retention and real-world application. Integration of multiple assessment methods, including patient-reported outcomes and objective behavioral measures, would provide more comprehensive evaluation of caring competencies. Investigation of optimal curricular timing, duration, and intensity would inform evidence-based curriculum design. Exploration of individual difference factors that predict intervention response could enable personalized educational approaches. For successful curricular integration and replication, medical schools should consider implementing this oncology psychology training through a tiered approach that begins with foundational psychological concepts embedded within existing pathophysiology courses, progresses to specialized modules during clinical rotations, and culminates in supervised patient interactions during oncology clerkships. Key implementation strategies include establishing partnerships with psychology departments or psycho-oncology services for specialized instruction, limiting class sizes to 15–20 students to ensure adequate discussion and personalized feedback, and scheduling the training during the transition from pre-clinical to clinical years when students possess sufficient medical knowledge but retain flexibility in professional identity development. Institutions seeking to replicate this model should ensure faculty development in emotional learning pedagogy, incorporate competency-based assessments of caring behaviors beyond traditional knowledge testing, allocate resources for standardized patients and video recording capabilities for communication practice, and establish longitudinal follow-up mechanisms to assess retention of caring behaviors in clinical practice. Additionally, international adaptations should consider local healthcare communication patterns and cultural attitudes toward emotional expression while maintaining the core components of experiential learning, reflective practice, and skills application that appear universally valuable for developing caring ability in medical students.

Several limitations warrant consideration. First, the quasi-experimental pre-post design (without a control group) prevents definitive conclusions about whether the course itself was associated with CAI improvements. Concurrent factors—such as students' clinical experiences during oncology rotations (e.g., interactions with distressed patients) or informal learning from supervisors—could have also contributed to the observed gains, as we cannot isolate the course's unique effect. Second, the small sample size limits the generalizability of our findings. The sample comprised postgraduate medical students at a single university, results may not apply to students at other institutions, those in undergraduate programs, or those with more diverse clinical backgrounds. Third, the study lacked long-term follow-up assessments, which is a critical limitation for evaluating the sustainability of caring ability improvements. Our data only capture immediate post-course changes (within 1 week of course completion), but healthcare education interventions ideally require 3–6 months follow-ups to assess whether skills are retained and applied in subsequent clinical practice. Fourth, the reliance on self-reported CAI scores introduces potential response bias. Students may have overestimated their caring ability post-course, rather than reporting their actual behaviors. Complementary objective measures—such as supervisor evaluations of caring behaviors or patient feedback—would have enhanced the validity of our findings. Finally, subgroup analyses should be interpreted cautiously. Small subgroup sizes reduce statistical power, increasing the risk of type II errors or overinterpreting minor differences as meaningful.

## Conclusions

Participation in the oncology psychology course was associated with meaningful improvements in caring ability among postgraduate medical students. These results support the potential value of integrating such courses into oncology training curricula, though future randomized controlled trials are needed to confirm whether the course itself causes these improvements. The differential responses across demographic subgroups highlight the importance of considering learner characteristics in educational planning. While limitations exist, these findings contribute to the growing evidence base supporting integrated psychosocial education in medical training and provide a foundation for future research in this critical area of healthcare professional development.

## Data Availability

The raw data supporting the conclusions of this article will be made available by the authors, without undue reservation.

## References

[B1] Avolio B. J. Reichard R. J. Hannah S. T. Walumbwa F. O. Chan A. (2009). A meta-analytic review of leadership impact research: experimental and quasi-experimental studies. Leadersh. Q. 20, 764–784. doi: 10.1016/j.leaqua.2009.06.006

[B2] Baile W. F. Buckman R. Lenzi R. Glober G. Beale E. A. Kudelka A. P. (2000). SPIKES-A six-step protocol for delivering bad news: application to the patient with cancer. Oncologist 5, 302–311. doi: 10.1634/theoncologist.5-4-302 10964998

[B3] Baron-Cohen S. Wheelwright S. (2004). The empathy quotient: an investigation of adults with Asperger syndrome or high functioning autism, and normal sex differences. J. Autism Dev. Disord. 34, 163–175. doi: 10.1023/B:JADD.0000022607.19833.00 15162935

[B4] Billings-Gagliardi S. Barrett S. V. Mazor K. M. (2004). Interpreting course evaluation results: insights from thinkaloud interviews with medical students. Med. Educ. 38, 1061–1070. doi: 10.1111/j.1365-2929.2004.01953.x 15461651

[B5] Brunero S. Lamont S. Coates M. (2010). A review of empathy education in nursing. Nurs. Inq. 17, 65–74. doi: 10.1111/j.1440-1800.2009.00482.x 20137032

[B6] Caruso R. Nanni M. G. Riba M. B. Sabato S. Grassi L. (2017). Depressive spectrum disorders in cancer: diagnostic issues and intervention. A critical review. Curr. Psychiatry Rep. 19:33. doi: 10.1007/s11920-017-0785-7 28488207 PMC5423924

[B7] Christov-Moore L. Simpson E. A. Coude G. Grigaityte K. Iacoboni M. Ferrari P. F. (2014). Empathy: gender effects in brain and behavior. Neurosci. Biobehav. Rev. 46, 604–627. doi: 10.1016/j.neubiorev.2014.09.001 25236781 PMC5110041

[B8] Cleland J. A. Abe K. Rethans J. J. (2009). The use of simulated patients in medical education: AMEE Guide No 42. Med. Teach. 31, 477–486. doi: 10.1080/01421590903002821 19811162

[B9] Cruess R. L. Cruess S. R. Boudreau J. D. Snell L. Steinert Y. (2014). Reframing medical education to support professional identity formation. Acad. Med. 89, 1446–1451. doi: 10.1097/ACM.0000000000000427 25054423

[B10] Day D. V. Fleenor J. W. Atwater L. E. Sturm R. E. McKee R. A. (2014). Advances in leader and leadership development: a review of 25 years of research and theory. Leadersh. Q. 25, 63–82. doi: 10.1016/j.leaqua.2013.11.004

[B11] Dekker J. Graves K. D. Badger T. A. Diefenbach M. A. (2020). Management of distress in patients with cancer-are we doing the right thing? Ann. Behav. Med. 54, 978–984. doi: 10.1093/abm/kaaa091 33416842 PMC7791612

[B12] Duberstein P. Meldrum S. Fiscella K. Shields C. G. Epstein R. M. (2007). Influences on patients' ratings of physicians: physicians demographics and personality. Patient Educ. Couns. 65, 270–274. doi: 10.1016/j.pec.2006.09.007 17125958

[B13] Dulay M. C. B. Domingo J. E. A. Domingo K. F. R. Domondon H. O. F. Dumangon L. G. Duran R. A. D. . (2018). An exploratory study of factors influencing student nurses' empathy. J. Nurs. Healthc. 3.

[B14] Epstein R. M. Hadee T. Carroll J. Meldrum S. C. Lardner J. Shields C. G. (2007). “Could this be something serious?” Reassurance, uncertainty, and empathy in response to patients' expressions of worry. J. Gen. Intern. Med. 22, 1731–1739. doi: 10.1007/s11606-007-0416-9 17972141 PMC2219845

[B15] Eva K. W. (2005). What every teacher needs to know about clinical reasoning. Med. Educ. 39, 98–106. doi: 10.1111/j.1365-2929.2004.01972.x 15612906

[B16] Fallowfield L. Jenkins V. (2004). Communicating sad, bad, and difficult news in medicine. Lancet 363, 312–319. doi: 10.1016/S0140-6736(03)15392-5 14751707

[B17] Fallowfield L. Jenkins V. Farewell V. Saul J. Duffy A. Eves R. (2002). Efficacy of a Cancer Research UK communication skills training model for oncologists: a randomised controlled trial. Lancet 359, 650–656. doi: 10.1016/S0140-6736(02)07810-8 11879860

[B18] Frenk J. Chen L. Bhutta Z. A. Cohen J. Crisp N. Evans T. . (2010). Health professionals for a new century: transforming education to strengthen health systems in an interdependent world. Lancet 376, 1923–1958. doi: 10.1016/S0140-6736(10)61854-5 21112623

[B19] Heaven C. Clegg J. Maguire P. (2006). Transfer of communication skills training from workshop to workplace: the impact of clinical supervision. Patient Educ. Couns. 60, 313–325. doi: 10.1016/j.pec.2005.08.008 16242900

[B20] Hu S. Chen J. Jiang R. Hu H. Hu Z. Gao X. . (2022). Caring ability of nursing students pre- and post-internship: a longitudinal study. BMC Nurs. 21:133. doi: 10.1186/s12912-022-00921-2 35644615 PMC9150307

[B21] Jarvis-Selinger S. Pratt D. D. Regehr G. (2012). Competency is not enough: integrating identity formation into the medical education discourse. Acad. Med. 87, 1185–1190. doi: 10.1097/ACM.0b013e3182604968 22836834

[B22] Kataoka H. U. Koide N. Ochi K. Hojat M. Gonnella J. S. (2009). Measurement of empathy among Japanese medical students: psychometrics and score differences by gender and level of medical education. Acad. Med. 84, 1192–1197. doi: 10.1097/ACM.0b013e3181b180d4 19707056

[B23] Kosik R. O. Fan A. P. Ren Y. Jiang B. Hsu Y. Li W. . (2018). Medical humanities education in China: an exploratory cross-sectional study. Lancet 392:S47. doi: 10.1016/S0140-6736(18)32676-X

[B24] Labrague L. J. McEnroe-Petitte D. M. D'Souza M. S. Hammad K. S. Hayudini J. N. A. (2020). Nursing faculty teaching characteristics as perceived by nursing students: an integrative review. Scand. J. Caring Sci. 34, 23–33. doi: 10.1111/scs.12711 31062401

[B25] Levinson W. Roter D. L. Mullooly J. P. Dull V. T. Frankel R. M. (1997). Physician-patient communication. The relationship with malpractice claims among primary care physicians and surgeons. JAMA 277, 553–559. doi: 10.1001/jama.1997.03540310051034 9032162

[B26] Mehnert A. Brahler E. Faller H. Harter M. Keller M. Schulz H. . (2014). Four-week prevalence of mental disorders in patients with cancer across major tumor entities. J. Clin. Oncol. 32, 3540–3546. doi: 10.1200/JCO.2014.56.0086 25287821

[B27] Mercer S. W. Reynolds W. J. (2002). Empathy and quality of care. Br. J. Gen. Pract. 52, S9–S12. 12389763 PMC1316134

[B28] Nekhlyudov L. Levit L. A. Ganz P. A. (2024). Delivering high-quality cancer care: charting a new course for a system in crisis: one decade later. J. Clin. Oncol. 42, 4342–4351. doi: 10.1200/JCO-24-01243 39356979 PMC11654450

[B29] Nkongho N. O. (2003). Assessing and Measuring Caring in Nursing and Health Sciences, 3rd Edition. Watson's Caring Science Guide 10: Caring Ability Inventory. doi: 10.1891/9780826195425.0010

[B30] Park E. M. Gelber S. Rosenberg S. M. Seah D. S. E. Schapira L. Come S. E. . (2018). Anxiety and depression in young women with metastatic breast cancer: a cross-sectional study. Psychosomatics 59, 251–258. doi: 10.1016/j.psym.2018.01.007 29525523 PMC5935568

[B31] Razavi D. Delvaux N. Marchal S. Durieux J. F. Farvacques C. Dubus L. . (2002). Does training increase the use of more emotionally laden words by nurses when talking with cancer patients? A randomised study. Br. J. Cancer 87, 1–7. doi: 10.1038/sj.bjc.6600412 12085247 PMC2364281

[B32] Stepien K. A. Baernstein A. (2006). Educating for empathy. A review. J. Gen. Intern. Med. 21, 524–530. doi: 10.1111/j.1525-1497.2006.00443.x 16704404 PMC1484804

[B33] Stewart M. Brown J. B. Donner A. McWhinney I. R. Oates J. Weston W. W. . (2000). The impact of patient-centered care on outcomes. J. Fam. Pract. 49, 796–804. 11032203

[B34] Stiefel F. Bourquin C. Layat C. Vadot S. Bonvin R. Berney A. (2013). Medical students' skills and needs for training in breaking bad news. J. Cancer Educ. 28, 187–191. doi: 10.1007/s13187-012-0420-6 23055132

[B35] Sung H. Ferlay J. Siegel R. L. Laversanne M. Soerjomataram I. Jemal A. . (2021). Global cancer statistics 2020: GLOBOCAN estimates of incidence and mortality worldwide for 36 cancers in 185 countries. CA Cancer J. Clin. 71, 209–249. doi: 10.3322/caac.21660 33538338

[B36] Thompson A. E. Voyer D. (2014). Sex differences in the ability to recognise non-verbal displays of emotion: a meta-analysis. Cogn. Emot. 28, 1164–1195. doi: 10.1080/02699931.2013.875889 24400860

[B37] Watson J. (2008). Nursing: The Philosophy and Science of Caring. Boulder, CO: University Press of Colorado.

[B38] Williams B. Brown T. McKenna L. Boyle M. J. Palermo C. Nestel D. . (2014). Empathy levels among health professional students: a cross-sectional study at two universities in Australia. Adv. Med. Educ. Pract. 5, 107–113. doi: 10.2147/AMEP.S57569 24833947 PMC4014368

[B39] Woodward V. M. (1998). Caring, patient autonomy and the stigma of paternalism. J. Adv. Nurs. 28, 1046–1052. doi: 10.1046/j.1365-2648.1998.00741.x 9840876

[B40] Xu J. (2008). Investigation on Caring Ability of Hospital Nurses (Master's thesis). Anhui Medical University, Hefei.

[B41] Zhang X. Pang H. F. Duan Z. (2023). Educational efficacy of medical humanities in empathy of medical students and healthcare professionals: a systematic review and meta-analysis. BMC Med. Educ. 23:925. doi: 10.1186/s12909-023-04932-8 38057775 PMC10698992

